# Acute Appendicitis in an 86-Year-Old Patient: Uncommon Age for a Common Disease

**DOI:** 10.7759/cureus.54957

**Published:** 2024-02-26

**Authors:** Moayad S Almusaylim, Mohammed S Foula, Jawad F Al Amin, Mohammad A Al-Abbad, Mustafa A Alsaleh

**Affiliations:** 1 College of Medicine, Imam Abdulrahman Bin Faisal University, Dammam, SAU; 2 Surgery, Imam Abdulrahman Bin Faisal University, Dammam, SAU; 3 General Surgery, Imam Abdulrahman Bin Faisal University, Dammam, SAU

**Keywords:** laparoscopic appendectomy, typical appendicitis in elderly, simple appendicitis, appendicitis in elderly, acute appendicitis

## Abstract

Appendicitis, an inflammation of the vermiform appendix, is one of the most common causes of acute abdomen and one of the most frequent indications for emergency abdominal surgery worldwide. Any person older than 65 years old is considered elderly. The elderly population constitutes only 5-10% of total appendicitis cases. The symptoms depend on the location of the appendix. Generally, lower abdominal pain and anorexia are known to be the most common symptoms of appendicitis. Although young adults have a higher prevalence of appendicitis, the elderly have a higher complication rate, 37.5% versus 43.97%. In this article, we report a case of appendicitis in an 86-year-old gentleman known to have type 2 diabetes mellitus, hypertension, and prostate cancer. The patient was managed successfully after a complicated hospital course and discharged in an improved and stable condition.

## Introduction

Appendicitis is an inflammation of the vermiform appendix. It is one of the most common causes of acute abdomen and one of the most frequent indications for emergency abdominal surgeries worldwide [1]. In clinical practice, treating the patient based on their age group is one of the important considerations clinicians should keep in mind. It is important to identify a patient’s age group to foresee how they may differ from the typical disease presentation. Elderly people are defined as any person aged 65 years or older [2]. Appendicitis in elderly patients accounts for 5-10% of total cases [3]. The prevalence of complicated appendicitis increases with age, reaching 37.5% in patients aged 40-64, 43.97% in those aged 65-74, and further rising to 56.84-63.0% for patients aged above 75 and 64.9-72.7% for those over 80 years old [4,5]. The most common symptom of acute appendicitis in the elderly is lower abdominal pain, followed by anorexia, nausea, and vomiting [4].

## Case presentation

An 86-year-old male patient presented to the emergency room complaining of right iliac fossa pain. The symptoms had started two days before his presentation, with pain located at the epigastric region which then shifted to the right iliac region. The pain was intermittent and increased on movement. It was sharp, did not radiate anywhere, was associated with nausea and vomiting, and once with food content. He denied any fever, urinary symptoms, and changes in bowel habits.

The patient had a past medical history of prostate cancer, currently on hormonal treatment. He was known to have type 2 diabetes mellitus and hypertension, controlled with medications. He was also a cardiac patient who had undergone percutaneous coronary intervention twice. He had undergone a right inguinal hernia open repair 17 years ago. The patient had a family history of colorectal cancer. On examination, the patient had elevated blood pressure of 156/124 mmHg, but other vital signs were within normal limits. The patient looked uncomfortable. However, he was alert, conscious, and oriented to time, place, and person. He was shivering and not in respiratory distress. Abdominal examination showed moderate right iliac fossa tenderness with positive rebound tenderness. Laboratory investigations showed leukocytosis, high neutrophil count, and elevated inflammatory markers. He also had hyperglycemia, with slightly decreased hemoglobin (Table 1).

**Table 1:** Laboratory investigations. HgB: hemoglobin; WBC: white blood cells; RBC: Red blood cells; CRP: C-reactive protein; LDH: lactate dehydrogenase

Laboratory test	Result	Normal range
HgB	12.9 g/dL	13.0–18.0 g/dL
WBC	13.9 × 10^3^/µL	4.0–11.0 × 10^3^/µL
Neutrophils	11.8 ×10^3^/µL	2–7.5 × 10^3^/µL
Lymphocytes	0.9 × 10^3^/µL	1–5 × 10^3^/µL
Platelets	228 × 10^3^/µL	140–450 × 10^3^/µL
RBC	4.08 × 10^6^/µL	4.70–6.10 × 10^6^/µL
CRP	10.80 mg/dL	0.10–0.50 mg/dL
LDH	427 U/L	125–220 U/L
Random blood glucose	188 mg/dL	70–140 mg/dL

**Figure 1:**
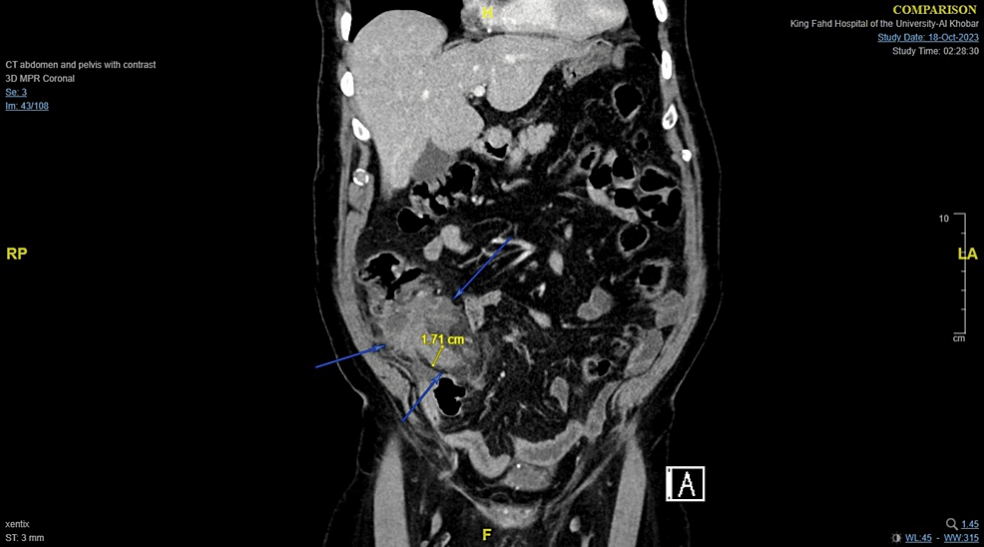
CT scan of the abdomen showing thickened dilated appendix reaching 1.71 cm with free fluid and fat stranding. The adjacent terminal ileum and cecum are significantly inflamed with enlarged regional lymph nodes.

The patient was started on intravenous (IV) Ringer’s lactate, analgesia, proton pump inhibitor (PPI), antiemetic, ciprofloxacin, and metronidazole. The patient was stabilized afterward. Electrocardiography (ECG) was unremarkable, and contrast-enhanced computed tomography (CT) scan of the abdomen and pelvis showed evidence of acute appendicitis (Figure 1).

The patient was diagnosed with acute appendicitis and underwent an emergency laparoscopic appendectomy. Intraoperatively, an inflammatory mass was noted at the right lower quadrant with gangrenous perforated midportion and a thickened but healthy base. Meticulous dissection using sharp and blunt techniques was done. The base of the inflamed appendix was closed by an endovascular gastrointestinal anastomosis stapler, and two drains were placed.

**Figure 2:**
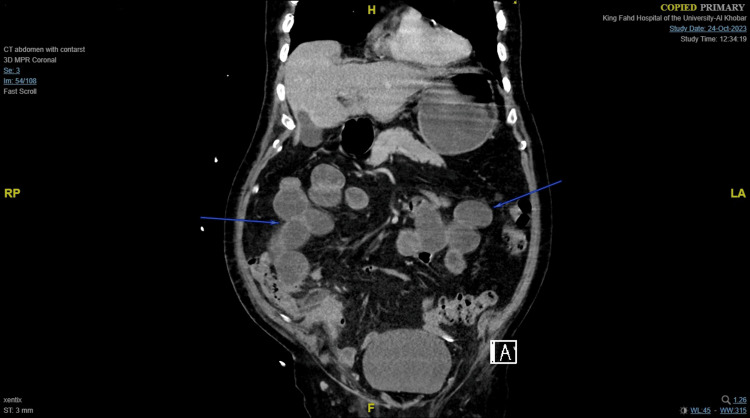
Follow-up contrast-enhanced CT scan of the abdomen on the sixth postoperative day showing fluid-filled dilated jejunal and ileal loops (reaching 4.8 cm) with no transition zone.

Postoperatively, the patient was kept on IV antibiotics, analgesia, PPI, and anticoagulants. A liquid diet was started on the first postoperative day, but he developed postoperative ileus one day later, and a nasogastric tube (NGT) was placed. A follow-up CT of the abdomen showed dilated bowel loops with no transition zone (Figure 2). The NGT was removed on the 11th postoperative day, and the patient was started on a soft diet which he tolerated well.

The patient was discharged on postoperative day 14 in good and stable condition. He was followed up in the clinic after 14 days with no active complaints. The final pathology report showed features consistent with perforated acute suppurative appendicitis.

## Discussion

Acute appendicitis is mainly caused by a luminal obstruction that can lead to inflammation, ischemia, and, consequently, perforation and peritonitis or contained abscess. The cause of obstruction differs according to age. At a young age, lymphoid follicular hyperplasia is the most common cause of obstruction of the vermiform appendix, whereas in older adults, fecolith, fibrosis, and neoplasia are the most common causes. When obstruction occurs, the intraluminal and intramural pressure of the appendix increases, leading to the occlusion of small vessels and resulting in ischemia [4]. Multiple risk factors for appendicitis have been reported in the literature, including unhealthy diet, smoking, a family history of appendicitis, low socioeconomic status, and the use of probiotics and antibiotics [6]. The typical symptoms of acute appendicitis include colicky pain that starts in the periumbilical area and then becomes constant and sharp, ultimately shifting to the right lower quadrant with tenderness at McBurney’s point. Anorexia, nausea, vomiting, and fever are associated symptoms [7]. The accuracy of the diagnostic evaluation relies on the experience of the attending physician. A high index of suspicion is crucial as elderly patients can present with atypical symptoms. A combination of clinical signs, laboratory findings, and imaging is important to establish a diagnosis. The laboratory tests for suspected acute appendicitis should include a complete blood count with differential and serum C-reactive protein, with their combination significantly increasing the sensitivity. Although there are many scoring systems for the diagnosis of acute appendicitis, the most common is the modified Alvarado score (Table 2). A score of ≤3 suggests that the diagnosis of appendicitis is unlikely and another diagnosis should be considered, while patients with a score of >3 should undergo further evaluation for appendicitis [8].

**Table 2:** Modified Alvarado score for the diagnosis of appendicitis.

Feature	Points
Migratory right lower quadrant pain	1
Anorexia	1
Nausea or vomiting	1
Tenderness in the right lower quadrant	2
Rebound tenderness in the right lower quadrant	1
Fever >37.5°C (>99.5°F)	1
Leucocytosis of white blood cell count >10 × 10^9^/L	2
Total	9

Contrast-enhanced CT of the abdomen is the imaging modality of choice while ultrasound and magnetic resonance imaging are reserved for children and pregnant women. The findings on CT can include appendiceal wall thickening or enhancement, peri-appendiceal fat stranding, and/or appendicolith [9].

The standard management of acute appendicitis is an appendectomy, either laparoscopic or open appendectomy. Recent studies suggest that conservative management with antibiotics is not inferior to surgical management in many cases [10]. However, for elderly patients in particular, a small study reported a 20% recurrence rate for those who were managed conservatively [11].

In elderly patients with appendicitis, the diagnosis may be challenging as the classical symptoms may not be prominent, or the presentation may be non-specific [4]. This could be because of some physiological changes in the elderly that can alter the response to the disease, such as diminished pain perception, localization, and decreased thermoregulation [12]. However, signs of peritonitis can be more pronounced [4]. Atypical presentations of appendicitis in the elderly include vague symptoms such as nausea, vomiting, generalized abdominal pain, and decreased oral intake [7,13]. Furthermore, they can present with changes in bowel habits in the form of diarrhea or constipation [14,15]. In addition, they can present with pain in atypical locations, such as left-sided abdominal pain or peri-umbilical abdominal pain radiating to the left side of the umbilicus [16,17].

Acute appendicitis in elderly patients is a challenging clinical entity, not only because of its challenging diagnosis, delayed presentation, and atypical clinical symptoms and signs but also because it is associated with a higher rate of complications such as perforation, gangrene, and abscess formation [10,18]. Exclusion of underlying malignancy is very important in this age group, especially cecal and appendicular tumors. Elderly patients with symptoms of acute appendicitis can harbor an underlying malignancy of the cecum or appendix, so it must be kept at the top of the differential diagnoses. As distinguishing appendicitis from colon cancer in elderly patients by symptoms or imaging may be challenging, a postoperative colonoscopy should be performed to exclude cancer [19]. Moreover, they may have a delayed recovery with prolonged hospital stays due to their concomitant diseases and reduced physiological reserve. Underlying medical conditions, such as congestive heart failure and diabetes, can increase the risk of complications and can affect the healing process. Moreover, elderly patients have reduced physiological reserve, which decreases their ability to withstand stress conditions such as surgical operations [4,20].

Our patient presented with the typical features of acute appendicitis including shifting pain, anorexia, nausea, and vomiting. His Alvarado score was 7. His CT scan showed a thickened dilated appendix with free fluid and fat stranding. During diagnostic laparoscopy, he had an inflammatory mass with perforated acute appendicitis. The operation was quite difficult, but no intraoperative complications occurred. However, he had a delayed recovery because of his prolonged postoperative ileus which may be due to his coexisting diseases and medications. The histopathological report confirmed acute suppurative appendicitis with no underlying malignancy.

We reviewed the literature to retrieve elderly patients with acute appendicitis. We included all patients above the age of 65 and identified a total of 13 cases. In those cases, abdominal pain and nausea were the most common symptoms. All cases were managed by appendectomy, and none of them were managed conservatively. The outcome was an uncomplicated recovery in seven cases, while four developed postoperative complications which were treated successfully. However, two patients developed multiorgan failure and died shortly after surgery (Table 3).

**Table 3:** Clinical data of elderly patients with appendicitis from published literature.

Number	Study	Age/Gender	Comorbidities	Risk factors for appendicitis	Symptoms	Management	Outcome
1	Su et al., 2011 [7]	79 years/Male	Unknown	No risk factors	Epigastric pain, nausea, vomiting, and distention	Appendectomy and abscess drainage	ICU admission for 5 days, shifted to the ward for 10 days, and discharged home
2	Lim et al., 2021 [13]	81 years/Male	Hypertension	No risk factors	Weakness, nausea, decreased oral intake, dizziness, fever, and abdominal pain	Laparoscopic appendectomy	Died after 4 days of operation
3	Najm et al., 2023 [14]	80 years/Male	Allergic asthma and NSAID intolerance	No risk factors	Diffused abdominal pain, asthenia, fecal vomiting, and constipation	Open appendectomy and abscess drainage	Uneventful recovery. Discharged home after 1 week
4	Sanda et al., 2011 [21]	75 years/Male	Parkinson’s disease and osteoarthritis	No risk factors	Abdominal pain and fever	Open appendectomy	Uncomplicated recovery
5	Okita et al., 2021 [15]	109 years/Female	Heart failure	No risk factors	Fever, diarrhea, and nausea	Open appendectomy and peritoneal lavage and drainage tube insertion	Discharged home after 15 days of operation
6	Singla et al., 2015 [16]	73 years/Male	Bronchiectasis and ischemic heart disease	No risk factors	Left-sided abdominal pain, constipation	Laparoscopic appendectomy	Recovery after treating postoperative intra-abdominal hematoma
7	Karaisli et al., 2018 [17]	69 years/Male	None	No risk factors	Periumbilical abdominal pain migrating to the left side of the umbilicus and nausea	Open appendectomy	Uncomplicated recovery
8	Ting et al., 2008 [22]	71 years/Male	Chronic obstructive pulmonary disease	No risk factors	Right upper quadrant pain radiating to the right iliac fossa	Laparoscopic appendectomy	Recovery after treating wound infection
9	Ahn et al., 2021 [23]	82 years/Male	Hypertension	No risk factors	Hematochezia	Laparoscopic partial cecectomy	Uncomplicated recovery
10	Gaisinskaya et al., 2022 [24]	97 years/Male	Dementia, benign prostatic hyperplasia, and urinary stones	No risk factors	Right-sided abdominal pain	Open bilateral inguinal hernia repair with mesh placement and appendectomy	Uncomplicated recovery
11	Guibentif et al., 2016 [25]	87 years/Male	Pulmonary tuberculosis	Antibiotic use	Fever and right lower quadrant pain	Laparoscopic appendectomy and abscess drainage	Favorable recovery
12	Peña et al., 2021 [26]	85 years/Female	Hypertension	No risk factors	Fever and epigastric abdominal pain radiating to both iliac fossae	Open appendectomy, abscess drainage, and antibiotics	Uneventful recovery. Discharged home after 1 week
13	De Souzaaet al., 2017 [27]	84 years/Male	Hypertension	No risk factors	Thigh pain, difficulty walking, and painful swelling of the right lower cx lateral abdomen	Open appendectomy, abscess drainage, and debridement	Continued to be hypotensive and developed organ failure. Died after 11 days of surgery

## Conclusions

Acute appendicitis in the elderly constitutes a small percentage of total appendicitis cases, has a wide range of presentations, and is associated with a higher rate of complications, making it a challenging diagnosis. Thus, we believe that acute appendicitis in the elderly needs to be further studied and more data should be added to the literature which will help establish the diagnosis of appendicitis to avoid delayed diagnosis and possible complications.
